# Two-Year Cohort Study of SARS-CoV-2, Verona, Italy, 2020‒2022

**DOI:** 10.3201/eid2904.221268

**Published:** 2023-04

**Authors:** Zeno Bisoffi, Nicoletta De Santis, Chiara Piubelli, Michela Deiana, Francesca Perandin, Pietro Girardi, Luca Heller, Natalia Alba, Carlo Pomari, Massimo Guerriero

**Affiliations:** Istituto di Ricovero e Cura a Carattere Scientifico Sacro Cuore Don Calabria Hospital, Verona, Italy (Z. Bisoffi, N. De Santis, C. Piubelli, M. Deiana, F. Perandin, C. Pomari, M. Guerriero);; AULDSS 9 Scaliger Regione del Veneto, Verona (P. Girardi, L. Heller, N. Alba)

**Keywords:** SARS-CoV-2, coronaviruses, viruses, coronavirus disease, COVID-19, pandemic, cohort study, prevalence, incidence, cumulative incidence, serologic testing, respiratory infections, reported data, sampling data, vaccine, elderly, frail persons, zoonoses, Verona, Italy

## Abstract

We performed a follow-up of a previously reported SARS-CoV-2 prevalence study (April‒May 2020) in Verona, Italy. Through May 2022, only <1.1% of the city population had never been infected or vaccinated; 8.8% was the officially reported percentage. Limiting protection measures and vaccination boosters to elderly and frail persons seems justified.

In Italy at the beginning of 2022, a large part of the population >10 years of age was vaccinated against SARS-CoV-2 (*1*). Nevertheless, a high number of new infections occurred in the following months, largely caused by increasing contagiousness of new virus variants. Reliable data on the proportion of the population that remains naive (unvaccinated and no history of infection) are crucial to improve SARS-CoV-2 infection control policies. Relying only on reported cases caused a gross underestimation of the true prevalence in the early stages of the pandemic, both in Italy (*2**–**5*) and elsewhere (*6**–**9*).

In April and May 2020, at the end of the first pandemic wave in Italy, we performed a prevalence survey on a random sample in Verona, Italy, and showed that ≈3% of the population had acquired the infection, 5 times the official figures (*4*). We then performed a follow-up of this cohort, ending May 31, 2022, to monitor the cumulative incidence of the infection and to estimate the proportion of the city population that had never had the infection or had been vaccinated, thus remaining fully susceptible or naive.

## The Study

**Figure 1:**
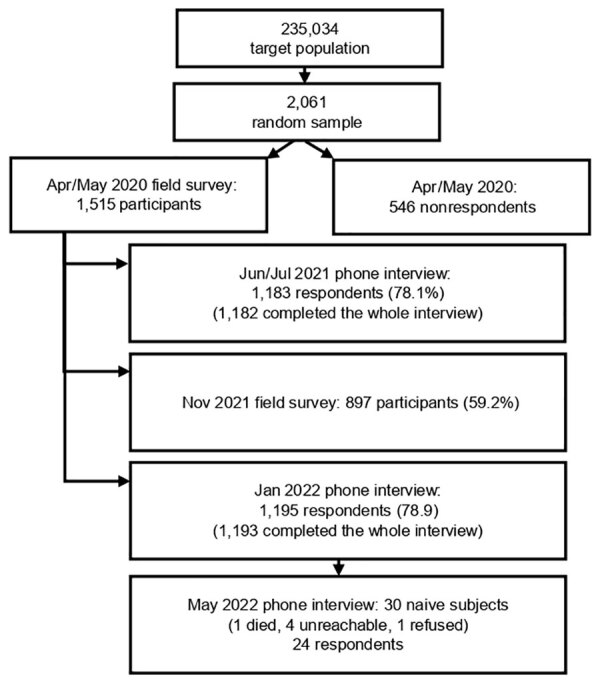
Flow chart for study of reported and sampling data for SARS-CoV-2, Verona, Italy, May 2020‒2022, starting from the initial sample of 1,515 persons who participated in the first study.

The study population has been described in detail (*4*). The initial cohort had 1,515 persons randomly selected from the city population (Figure 1; Appendix Figure). Mean age was 49.1 years, and most (54%) persons were women. Ten (0.7%) persons were positive for SARS-CoV-2 RNA, 40 (2.6%) were positive for IgG against nucleocapsid protein of SARS-CoV-2, and 1,465 (96.7%) tested negative. Using latent class analysis, we estimated a 3% prevalence of infection (*4*). We also summarize follow-up studies of the initial cohort (Figure 1).

We performed 3 follow-up surveys. The first survey was a telephone survey during June‒July 2021. The second survey, during November 2021, was in-person interviews on previous infections and vaccination status, and molecular (reverse transcription PCR) and antibody testing. The third survey was a telephone survey during January 2022.

**Figure 2:**
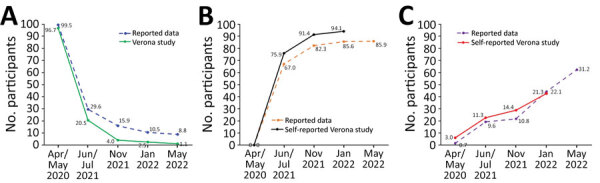
Reported and sampling data for SARS-CoV-2, Verona, Italy, May 2020‒2022. Comparison is shown between official (reported) and Verona study proportions of naive (A), vaccinated (B), and infected (C) persons. Values along data lines indicate cumulative incidence.

On May 31, 2022, those persons who were still naive in January were interviewed again. Survey data were then compared with report data from the city’s health authority (Figure 2).

During June‒July 2021, of the initial cohort of 1,515 persons, 1,182 (78.0%) responded, of whom 134 (11.3%) reported having had SARS-CoV-2 laboratory-confirmed infection at least once. Of those who had been vaccinated (897, 75.9%), a total of 563 (62.8%) had already received the second dose. A total of 242 (20.5%) persons did not reported vaccination or previous infection.

During November 2021, a total of 897 persons (59.2% of the initial cohort) consented to participate. All were administered a questionnaire, and we obtained nasal and pharyngeal swab specimens and blood samples from all consenting persons. We performed reverse transcription PCR of swab specimens as described (*4*) to detect active infections; only 1 (0.1%) specimen showed a positive result.

We analyzed serum samples by using the SARS-CoV-2 IgG-N Assay (Abbott, https://www.ie.abbott) to detect IgG against nucleocapsid protein, as described (*4*). We also used the SARS-CoV-2 IgG II Quant Assay (Abbott) for the quantitative measure of IgG against spike (receptor-binding domain) protein according to the manufacturer’s procedure by using the ARCHITECT I System (Abbott). We also performed this test on biobank samples from the initial 2020 cohort, when the test was not yet available, to make it possible to compare 2020 results with 2021 results.

We compared the results of antibody tests from the survey with 2020 data (Table). A total of 160 (17.8%) of 897 persons tested positive for nucleocapsid IgG, (which is unaffected by vaccination), and 831 (92.7%) of 896 persons (1 missing value) tested positive for antibody against spike (receptor-binding domain) protein, which reacts to vaccination and natural infection. Of the 34 persons who had tested positive for nucleocapsid IgG during 2020, half had negative results at the following survey.

**Table:** Comparison of antibody test results in 2 field surveys for SARS-CoV-2, Verona, Italy, May 2020 and November 2021

Characteristic	2021			
	Negative, no. (%)	Positive, no. (%)	Total, no. (%)	p value
IgG against nucleocapsid				
2020				<0.0001
Negative, no. (%)	720 (83.4)	143 (16.6)	863 (100.00)	
Positive, no. (%)	17 (50.0)	17 (50.0)	34 (100.0)	
Total, no.(%)	737 (82.2)	160 (17.8)	897 (100.0)	
IgG against spike protein				
2020				
Negative, no. (%)	65 (7.6)	796 (92.4)	861 (100.0)	
Positive, no. (%)	0 (0)	35 (100.0)	35 (100.0)	0.170*
Total, no. (%)	65 (7.3)	831 (92.7)	896 (100.0)	

*By Fisher exact test.

At the interview, of the 897 persons, 820 (91.4%) reported being vaccinated, of whom 735 (89.6%) had already received their second dose; 128 (14.3%) reported being infected at least 1 time. There were only 36 (4.0%) naive persons (no antibodies and no history of infection or vaccination).

During January 2022, of the 1,193 persons (78.7% of the initial cohort) who responded, 254 (21.3%) reported previous infections, and 1,123 (94.1%) had been vaccinated, including 322 (28.7%) with 2 doses and 764 (68.0%) with 3 doses. A total of 36 (3.0%) reported no infection or vaccination, of whom 6 were antibody positive in the previous survey. We classified the remaining 30 (2.5%) persons as naive and listed them for another interview on May 31, 2022.

During May 2022, of the 30 persons from the previous survey, 1 had died, 4 were unreachable, and 1 refused to answer. Of the remaining 24 persons, 8 were infected and remained unvaccinated, 2 were vaccinated and not infected, and 1 was vaccinated and infected. A total of 13 (1.1%) of 1,187 persons presumably remained naive. Thus, at the end May 2022, the best estimate from the study population was that 98.9% of persons >10 years of age in Verona had been infected, vaccinated, or both.

We compared data resulting from the random sample analysis (Figure 2) with reported data available up to May 31, 2022. The reported initial prevalence was much lower; the data had practically coincided in January 2022.

During May 2022 the cumulative reported incidence reached 31.2%, and the proportion of naive population was 8.8%, versus the 1.1% we found in our study (Figure 2). The actual percentage might be even lower, considering that results for IgG against nucleocapsid tend to become negative over time. Thus, we might have failed to detect some previous infections in the last field survey and, in any case, we did not perform further antibody investigations in the later stages.

## Conclusions

According to the survey data, almost the entire population of Verona had some degree of protection in May 2022 against the severe forms of the disease. After a natural infection, the risk for severe forms of COVID-19 is much attenuated, even for nonvaccinated persons (*10**–**13*). The 3 doses of vaccine confer a long-term protection against severe disease, and hybrid immunity is more effective (*14*). Because immunity tends to wane over time, our finding that almost the whole population had been infected or vaccinated does not mean that all persons are protected. Moreover, immunity against new infections is much less effective and is short lasting, especially for the Omicron variants (*11*,*15*). The high contagiousness of these variants will predictably lead to continued circulation of the virus, which will act as a booster for most persons.

The first limitation of this study is that a not negligible proportion of the initial cohort were not able to be followed up. A selection bias cannot be excluded because persons who participated in the follow-up might be more health-conscious and more likely to adhere to vaccination. Only in November 2021 was it possible to repeat the molecular and serologic study. However, even with those limitations, we were able to reconstruct the trend of the pandemic in Verona and compare research results with reported data. Our estimate of the population that is still completely naive is lower than the official figures.

In Verona, the pandemic seems to have entered a phase in which we can be cautiously optimistic about its future course. It remains crucial to protect the frail and elderly persons, including those given booster vaccinations when indicated, but a cautious relaxation of restrictions for the general population seems justified, and repeated boosters for nonfrail persons might not be necessary.

Appendix Additional information on 2-year cohort study of SARS-CoV-2, Verona, Italy, 2020‒2022.
