# Supplementary material for: Two-Year Cohort Study of SARS-CoV-2, Verona, Italy, 2020‒2022
Source: Emerg Infect Dis. 2023 Apr;29(4):822–5. doi: 10.3201/eid2904.221268 (PMC10045681; doi:10.3201/eid2904.221268)
Supplement: Appendix — Additional information on 2-year cohort study of SARS-CoV-2, Verona, Italy, 2020‒2022. [file 22-1268-Techapp-s1.pdf]

Article DOI: https://doi.org/10.3201/eid2904.221268

Two-Year Cohort Study of SARS-CoV-2, Verona, Italy, 2020–2022

Appendix.

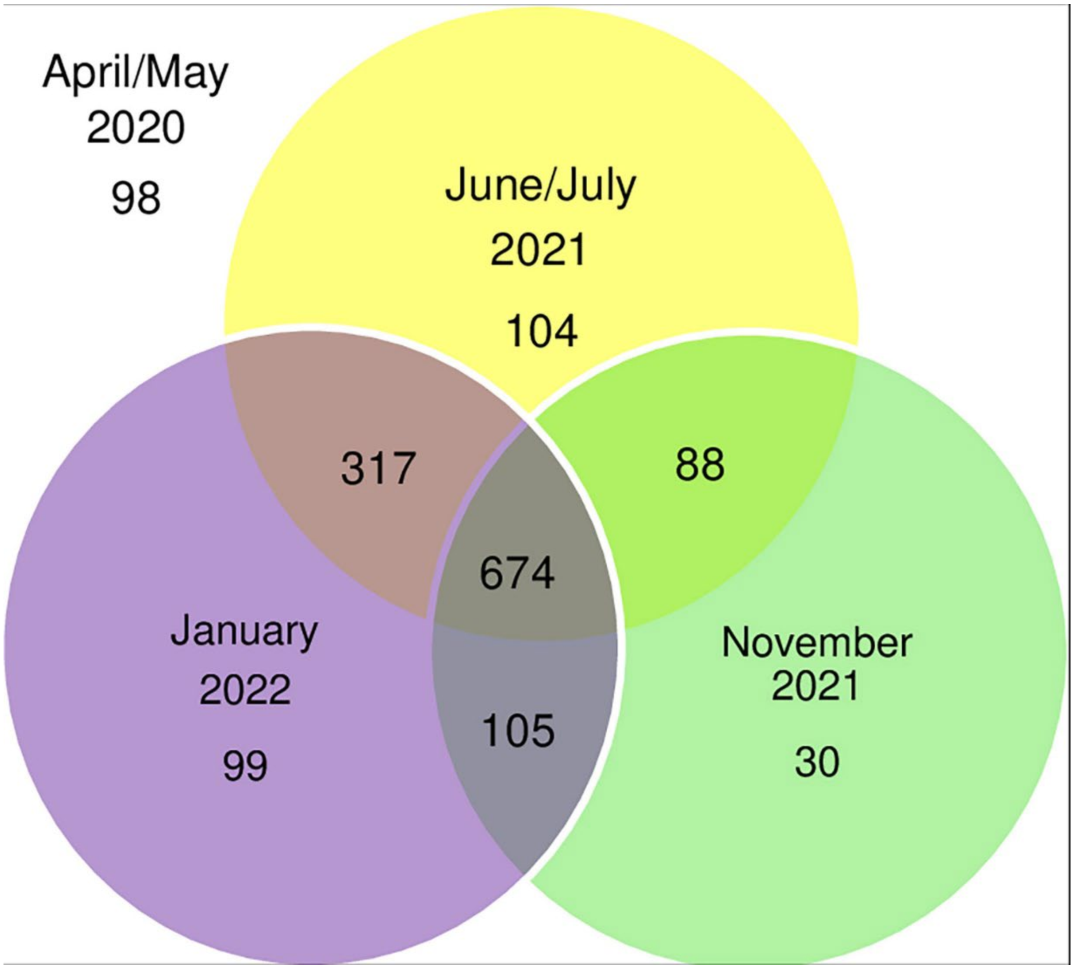

Appendix Figure. Eulero-Venn representation of the 3 main phases of the study, showing that 674 persons participated in all 3 phases.
